# 

**Published:** 1850-07

**Authors:** 


### CONTENTS

OF THE

### MEDICAL EXAMINER.

NEW SERIES—NO. LXVII.—JULY, 1850.

### ORIGINAL COMMUNICATIONS.

Pathological Cases. By Carter P. Johnson, M. D., Professor of Anatomy and Physiology in the Medical Department of Hampden Sidney College, Richmond, Virginia, - - - 379
On the use of Strychnia in Intermittent Fever. By S. E. McKinley, M. D., of Williamsport, Tenn., - - - - - 382
Surgical Cases. Reported by Charles Sutherland, M. D., late Assistant to the Clinic of Jefferson Medical College, - - 384
Observations on Endo-Cardial Coagula. By Marcus Dana, M. D., of Seneca County, Ohio, - - - - - - 389

### BIBLIOGRAPHICAL NOTICES.

Ship Fever, so-called: its History, Nature, and best Treatment. The Fiske Fund Prize Dissertation for 1849. By Henry Grafton Clark, M. D., Member of the Boston Society for Medical Improvement, - - - - - - - - 397

Report of the Committee of Internal Health on the Asiatic Cholera, together with a Report of the City Physician of the Cholera Hospital. Boston, 1849, - - - - - - 399
A Memoir on Stricture of the Urethra. By J. P. Mettauer, M. D., LL. D., Professor of the Principles and Practice of Surgery in the Medical Department of Randolph Macon College, Va., - 404
Southern Medical Reports: Consisting of General and Special Reports of the Medical Topography, Meteorology and Prevalent Diseases in the following States: Louisiana, Alabama, Mississippi, North Carolina, South Carolina, Georgia, Florida, Arkansas, Tennessee and Texas; to be published annually. Edited by E. D. Fenner, M. D., of New Orleans, Member of the American Medical Association, - - - - - 406

### EDITORIAL.

American Medical Association, - - - - - 423
Medical Appointments, - - - - - - 424
Death of Dr. Amos Twitchell, - - - - - 424
Assimilated Rank in the Navy, - - - - - 424
Astley Cooper Prize, - - - - - - 425
Correspondence between Dr. C. J. B. Williams and a Homœopathic Practitioner, - - - - - - - 426
Deaths in Philadelphia from April 20th to June 22d, 1850. Reported by Mr. James Aitken Meigs, Student of Medicine, - - 427
Death of Dr. Robert Egglesfeld Griffith, - - - - 428
Medical Department of the Army, - - - - - 428

### RECORD OF MEDICAL SCIENCE.

### ANATOMY AND PHYSIOLOGY.

Impulse of the Healthy Heart. By O'Bryen Bellingham, M. D., - 429
Researches on the Development of the Muscular Fibres of the Heart. By Dr. Lebert, - - - - - - - 433

### PATHOLOGY AND PRACTICE OF MEDICINE.

Cases of Poisoning by Colored Confectionery, with Remarks. By H. Letheby, M. B., Lecturer on Chemistry in the London Hospital, - - - - - - - - - 434

MATERIA MEDICA AND THERAPEUTICS.

New Anti-Spasmodic—Sumbul, - - - - - 437

SURGERY.

On Excision of the Os Calcis, in incurable Diseases of that Bone, as a substitute for Amputation of the Foot. By W. B. Page, Esq., Surgeon to the Cumberland Infirmary, - - - 438

